# A Visual-Based Approach for Indoor Radio Map Construction Using Smartphones

**DOI:** 10.3390/s17081790

**Published:** 2017-08-04

**Authors:** Tao Liu, Xing Zhang, Qingquan Li, Zhixiang Fang

**Affiliations:** 1 Shenzhen Key Laboratory of Spatial Smart Sensing and Services, Shenzhen University, Shenzhen 518060, China; liuzimo@whu.edu.cn (T.L.); liqq@szu.edu.cn (Q.L.); 2 Key Laboratory for Geo-Environment Monitoring of Coastal Zone of the National Administration of Surveying, Mapping and Geoinformation, Shenzhen University, Shenzhen 518060, China; 3 State Key Laboratory of Information Engineering in Surveying, Mapping and Remote Sensing, Wuhan University, Wuhan 430079, China; zxfang@whu.edu.cn

**Keywords:** indoor localization, radio map, smartphone, structure from motion, fingerprint

## Abstract

Localization of users in indoor spaces is a common issue in many applications. Among various technologies, a Wi-Fi fingerprinting based localization solution has attracted much attention, since it can be easily deployed using the existing off-the-shelf mobile devices and wireless networks. However, the collection of the Wi-Fi radio map is quite labor-intensive, which limits its potential for large-scale application. In this paper, a visual-based approach is proposed for the construction of a radio map in anonymous indoor environments. This approach collects multi-sensor data, e.g., Wi-Fi signals, video frames, inertial readings, when people are walking in indoor environments with smartphones in their hands. Then, it spatially recovers the trajectories of people by using both visual and inertial information. Finally, it estimates the location of fingerprints from the trajectories and constructs a Wi-Fi radio map. Experiment results show that the average location error of the fingerprints is about 0.53 m. A weighted k-nearest neighbor method is also used to evaluate the constructed radio map. The average localization error is about 3.2 m, indicating that the quality of the constructed radio map is at the same level as those constructed by site surveying. However, this approach can greatly reduce the human labor cost, which increases the potential for applying it to large indoor environments.

## 1. Introduction

With the great increment of mobile devices (e.g., smartphones), people now pay more attention to mobile navigation and location-based services. While the global positioning system (GPS) is widely used outdoors, indoor navigation remains a challenge due to the lack of an accurate, low-cost and widely available indoor localization solution. Nowadays, the commonly used indoor localization technologies include Wi-Fi [1], Bluetooth [2], magnetic fields [3], ultrasound [4], radio-frequency identification (RFID) [5], Ultrawide Band (UWB) [6], and so on. In particular, a Wi-Fi fingerprinting-based solution has attracted significant attention since it takes advantage of the existing Wi-Fi infrastructures (e.g., 802.11 Wi-Fi infrastructures) and mobile devices (e.g., smartphones). There are typically two phases for WiFi fingerprinting: the offline phase and the online phase. During the offline phase, the location-dependent received signal strength (RSS) from multiple Wi-Fi access points (APs) is collected to construct a fingerprint database (i.e., radio map). During the online phase, the location of a mobile user is determined by matching the instantaneous RSS with the fingerprints in the radio map.

The constructing and maintaining of RSS radio map is very important for WiFi fingerprinting based indoor localization systems. However, this process is quite laborious, expensive and time-consuming, especially if the environment is a large size (e.g., shopping mall or supermarket). It is an unavoidable bottleneck that limits the potential of this indoor localization approach for large-scale commercial use. Therefore, it is extremely important to propose solutions that can reduce the cost and workload required for radio map construction.

Much effort has been devoted to reducing the intensive costs of manpower and time for radio map construction. Many researchers [7,8] have tried to replace the site survey process by the use of wireless radio propagation models. Recently, various research works have focused on using rigorous deterministic radio propagation techniques based on ray tracing to generate the fingerprinting database in many occasions. These studies achieved good localization results and significantly reduced the workload needed for site surveying. However, one problem is that the location of Wi-Fi APs is required to be previously known, which may be difficult for many large indoor environments (e.g., shopping malls). Other researchers [9,10,11] have focused on developing zero-configuration indoor localization systems that do not require an explicit site survey or an offline phase, but instead implement the training phase during the use of the system. In general, although these systems can be directly applied without site surveying, they still cannot provide reliable localization results before the initialization and training phase are finished.

With the development of wireless and embedded technology, most smartphones are now equipped with various built-in sensors, such as cameras, accelerometers, gyroscopes, and electronic compasses, which provide a suitable interface for sensing and collecting information about indoor spaces. Nowadays, people spend most of their time (over 80%) in indoor environments. If people are able to participate in a site survey with smartphones and contribute their data to the construction of the radio map, the burden of location fingerprinting can be significantly reduced. By using the built-in sensors of smartphone, the collected data can be employed to estimate the trajectory of people and generate WiFi fingerprints for radio map construction. Many researchers [12,13,14] have proposed methods that connect independent fingerprints to radio maps by leverage user motions. Most of these methods use a pedestrian dead reckoning (PDR) technique for restoring the indoor trajectories of pedestrians. However, the accurate calculation of heading angle remains the most challenging problem in PDR. Most PDR systems calculate heading angle by using magnetometers or gyroscopes. However, magnetometer-based heading calculation is difficult in indoor environments because magnetometers are strongly influenced by magnetic perturbations produced by manmade infrastructure such as metal structures, electronic equipment or power lines. In addition, due to the drift noise of Micro-Electro-Mechanical System (MEMS) gyroscopes, the error of heading calculation accumulates as time goes on, which significantly affects the quality of the constructed radio map.

This study developed a visual-based approach for radio map construction based on the integration of both visual and inertial information. The main idea of this method is to extract fingerprints from the precisely restored walking trajectories and connect the fingerprints to radio maps. To better estimate the location of fingerprints, visual data (collected by smartphone camera) is employed to calculate the azimuth of trajectories. An SFM (Structure from Motion) based algorithm is proposed to estimate heading angle and recover the trajectories based on a multi-constrained image matching method. Gyroscope data is also employed to increase the robustness of heading estimation. A radio map can be constructed based on the calibrated fingerprints, which can greatly reduce the human labor needed for site surveying. This visual-based approach can be used to collect radio maps in different types of indoor environments, such as corridor-like spaces, room-like spaces as well as wide spaces. The turning angle of mobile users can be arbitrary, and there is no constraint for their walking behavior or turning activities. Indoor maps are also not needed for this approach, which can increase its potential for practical use.

The remainder of this paper is organized as follows. Section 2 provides a literature review. Section 3 describes the methodology of the proposed approach. The experimental results and analysis are described in Section 4. Section 5 concludes the paper.

## 2. Related Work

A Wi-Fi fingerprinting-based indoor localization method is welcomed by the majority of commercial customers, for their commonly used Wi-Fi infrastructure, convenient localization type and reliable positioning accuracy. The main idea of fingerprinting-based indoor localization is to utilize the difference of multi-source signals strength to distinguish location in indoor area. It always contain two modules: the first module is to fingerprint the surrounding signatures at the location of each sampling point in indoor areas and then build a fingerprint database (i.e., radio map). The second module is to estimate location through comparing the real-time RSS observation against that stored in the database. A lot of research concentrates on fingerprinting-based techniques for indoor localization. RADAR [1] is an early fingerprinting-based system proposed by Microsoft Research. The mean value of RSS at each sampling point is recorded in a radio map. Horus [15] improved upon RADAR by employing probabilistic techniques, which use the mean value and standard deviation of RSS as fingerprints, based on maximum likelihood method. Similar works are described in [16,17], which use probabilistic techniques for fingerprinting-based indoor localization. Park et al. [18] proposed an organic location system, which used a Voronoi diagram method for conveying uncertainty and a cluster-based method to discard erroneous user data. Au et al. [19] clustered RSS fingerprints after building ra adio map and used the compressive sensing theory to solve the positioning problem. All of these indoor localization approaches require a site survey process to construct radio maps of indoor areas. The main limitation of fingerprint-based methods is the extensive workload needed for radio map collecting and calbrating.

Another scheme for indoor localization is to use the inertial sensor based self-contained technique. Dead reckoning (DR) systems use inertial sensors such as accelerometers and gyroscopes to estimate user location. The main idea of DR is to derive one’s current location by adding the estimated displacement to the previously estimated one. The localization result from DR-based navigation system is always-available and is independent from external infrastructures. It is widely used in various smartphone-based tracking and localization studies. In [20], several methods were used to detect steps and estimate travelled distance based on acceleration data. The average error rage of step detection on various walking patterns was about 2.925%, indicating that the step number could be precisely estimated by using smartphones. The major drawback of PDR is that the location error will accumulate as distance traveled increases. To solve this problem, some of the research [21,22,23,24,25,26] aimed to restrict the accumulative error of PDR for indoor localization. The activity based map matching method was utilized to eliminate the cumulative error of PDR [21,22,23]. These methods need to recognize user’s activities and match their activities to the corresponding specific points (e.g., elevator) in indoor maps. The system proposed in [24] used RFID tags in indoor environments to recalibrate the accumulative errors. In [25], a PDR/Wi-Fi integrated indoor localization approach was proposed by using a Kalman filter. In [26], human activities were matched with road networks to correct the accumulative error of PDR by using a Hidden Markov Model. Most of these methods need external infrastructure or prior knowledge of the environment, which increases the difficulty of applying these methods to practical applications.

Visual data is another potential information source that can be used for indoor localization. For example, the computation of ego-motion is an important problem in autonomous navigation, which can be stated as the recovery of observer rotation and direction of translation using monocular or stereo sequences [27,28,29,30]. In addition, ego-motion estimation methods have also been applied to smartphone-based applications. For example, an ego-motion estimation algorithm was developed for augmented reality (AR) applications using Android smartphones [31].They ported the Parallel Tracking and Mapping (PTAM) [32] algorithm to locate the smartphones and used an Extended Kalman Filter (EKF) to smooth the trajectory estimates given by the PTAM. In [33], an egocentric motion tracking method was employed to recognize hand gestures for smartphone-based AR or Virtual Reality (VR) using single smartphone monocular rear-camera. There are also monocular ego-motion systems that combine Inertial Measurement Unit (IMU) and cameras (in mobile devices) for indoor mapping and blind navigation [34,35,36]. In [37,38], a heading change detection method was proposed by calculating the vanishing points in consecutive images. The performance of this method highly depends on the number of lines found in images and cannot be used to estimate the heading change of sharp turns. As a well-known imaging technology, the SFM method can used to recover the relative camera pose and 3D structure from a set of camera images. It has been used for planetary rovers by the NASA Mars exploration program [39]. In [40], iMoon built a 3D model of indoor environments for indoor navigation by using SFM technology. In [41], an image-based localization approach was proposed based on a probabilistic map by using 3D-to-2D matching correspondences between a map and a query image. Some studies [42,43] have tried to use an SFM method to estimate the trajectory of a moving camera. However, the image-based systems achieve indoor localization by returning the location of a query image, which makes it difficult to provide continuous positioning information. In addition, the mismatching problem (i.e., false matches of images) may also decrease the accuracy of image-based indoor localization.

In summary, the collected visual data from smartphones is helpful for restoring walking trajectories. Visual information has the potential to improve the performance of heading angle estimation. In this study, a visual-based approach is proposed that integrates both visual and inertial information to accurately estimate user trajectories. A multi-constrained image matching method is designed to improve the performance of trajectory reconstruction. By extracting WiFi fingerprints from spatially estimated trajectories, this visual-based approach can automatically construct indoor radio maps, which may significantly reduce the human labor needed for site surveys.

## 3. Methodology

**Figure 1:**
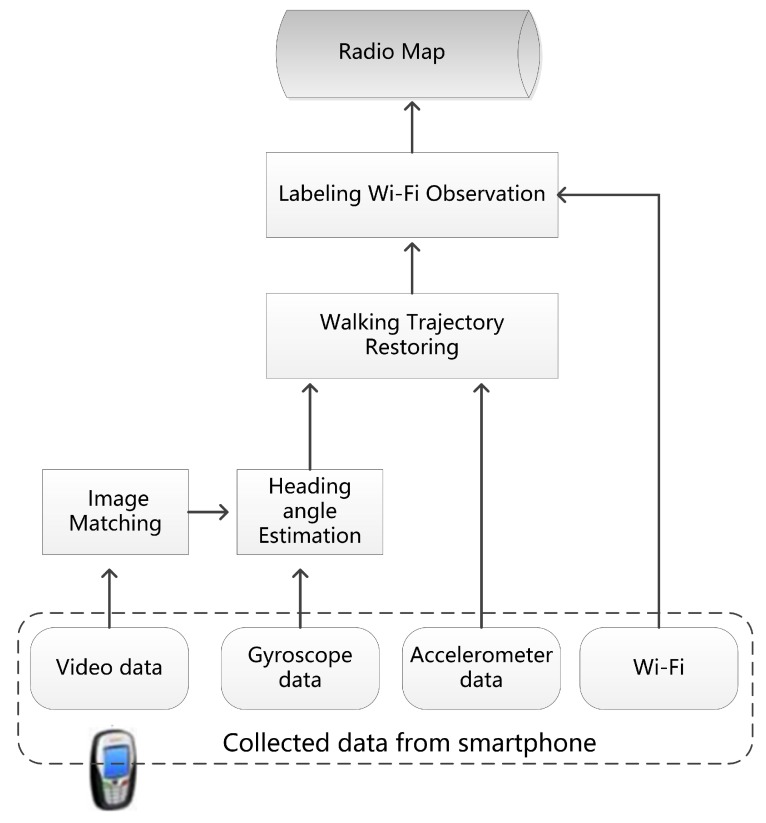
The overview of this proposed method.

The overview of this approach is described in Figure 1. This approach uses the built-in sensors of a smartphone to collect sensor data, including video frames, WiFi signals and inertial readings. During the data collection, a user holds a smartphone in his/her hand in front of the body (keep the camera forward facing and maintain the posture) and walks normally in indoor areas. The turning angle of the user can be arbitrary, and there is no constraint for their turning activities. To improve the location accuracy of WiFi fingerprints, this approach integrates both visual and inertial information to estimate the heading angle of trajectories. The SFM method is employed to estimate the heading angle by using video frames. A multi-constrained image matching method is designed to improve the performance of the SFM method. In addition, the readings from a smartphone MEMS gyroscope are also used to improve the robustness of heading angle estimation. After the trajectories are spatially estimated, WiFi fingerprints can be extracted to generate indoor radio maps.

### 3.1. Multi-Constrained Image Matching

Image matching technology is used to find the correspondence between two or more images on the pixel scale. Taking advantage of the correspondence among pixels, it is able to infer the relationship between each pair of adjacent images from video frames. Currently, there are various image matching methods. Most of these methods need to detect distinctive and invariant features from images, which are important for establishing the correspondence among pixels. Scale invariant Feature Transform (SIFT) [44] is one of the most popular image feature in the computer version, which is invariant to rotation, translation and scale variation between images and partially invariant to affine distortion, illumination variance and noise [45]. The main idea of the SIFT feature is to calculate the difference of gradient magnitude and orientation on multi-scale Gaussian space, counting the weighted gradient magnitude orientation histogram of the keypoint. It use a 128-dimension vector to express the keypoint descriptor.

The multi-constrained image matching method first extracts the SIFT feature and keypoint descriptor from the collected video frames. Image points are matched by individually comparing each feature descriptor. There are many metrics of similarity measurement of vector, including Euclidean distance, Manhattan distance, correlation coefficient, etc. However, the false matching between images cannot be eliminated if only these metrics are used. In order to remove the false matching results, three constraints are used in this method:

1. Ratio constraint. For a keypoint $P_{0}$ from image *a*, its best matching point from image *b* can be calculated as: $d_{i}=\sqrt{\sum_{j=1}^{128}{\left(v_{j}-v_{j}^{{}^{\prime }}\right)}^{2}}$ , where *v* is the descriptor vector of $P_{0}$, $v^{{}^{\prime }}$ is the descriptor vector of keypoints $P_{i}$ from image *b*, *j* is the dimension of the SIFT feature vector, $d_{i}$ is the Euclidean distance between feature vectors. The ratio constraint means that if the ratio of the smallest $d_{1}$ to the second smallest $d_{2}$ is lower than a threshold *r*, the keypoint $P_{i}$ is treated as a candidate for the best matching keypoint of $P_{0}$.
2. Symmetry constraint. For a pair of images, it is possible that a keypoint from image *a* may be matched with multiple keypoints in image *b*. The symmetry constraint is used to eliminate this type of false match. Each pair of adjacent images is matched to each other two times: (1) the keypoints from image *a* are matched to the keypoints from image *b*; and (2) after that, the keypoints from image *b* are matched to the keypoints from image *a*. The final keypoint pairs of the two images must be the common parts of the two times of matching.
3. RANSAC constraint. Random sample consensus (RANSAC) is an iterative method used to estimate parameters of an estimation model from a set of observed data that contain inliers and outliers [46]. We use four pairs of matching points to compute the homography matrix that can describe the translation, rotation, affine and other coordinate transformation. Using the homography matrix and the coordinates of matching points, the coordinate conversion error and the outliers can be calculated by iterating this method until obtaining the homography matrix with the maximum number of inliers. The performance of the image matching can be improved after the outliers are removed.

**Figure 2:**
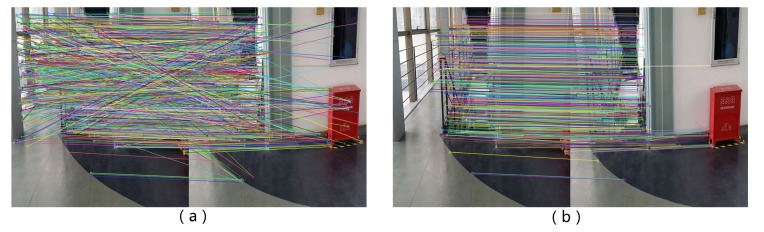
The matching results of SIFT and the multi-constrained algorithm. (**a**) the matching result of the SIFT method; (**b**) the matching result of the proposed method.

By employing the three constraints, the result of image matching can be improved. An example is shown in Figure 2, where the mismatchings of the two images are obviously reduced after these constraints are considered. Based on the multi-constrained image matching, the SFM method can be used for heading angle estimation.

### 3.2. SFM-Based Heading Angle Estimation

**Figure 3:**
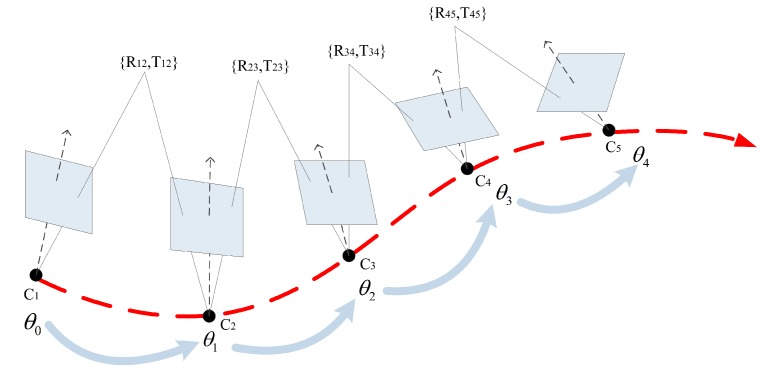
The details of the SFM-based heading angle estimation method.

The schematic diagram of the SFM-based heading angle estimation method is shown in Figure 3. In SFM, the matching results of two adjacent images are used to calculate the fundamental matrix *F* based on the epipolar geometry of two camera poses. Before the SFM process, the smartphone camera is calibrated based on the Matlab Camera Calibrator (Matlab 8.x on Windows) [47] , which can be used to estimate the parameters of the intrinsic matrix .The fundamental matrix *F* can be calculated by a set of homogeneous image keypoints:

(1) $$\left[\begin{array}{ccc} u_{i}^{\prime } & v_{i}^{\prime } & 1 \end{array}\right]\left[\begin{array}{ccc} f_{11} & f_{12} & f_{13}\\ f_{21} & f_{22} & f_{23}\\ f_{31} & f_{32} & f_{33} \end{array}\right]\left[\begin{array}{c} u_{i}^{\prime }\\ v_{i}^{\prime }\\ 1 \end{array}\right]=0,$$

where $m_{i}$($u_{i}$, $v_{i}$, 1)${}^{T}$, $m_{i}^{\prime }$($u_{i}^{\prime }$, $v_{i}^{\prime }$, 1) are the homogeneous keypoints of the matched keypoint set {$m_{i}$, $m_{i}^{\prime }$|*i* = 1, 2, … n}. Given eight or more pairs of matched keypoints, it is possible to linearly solve matrix *F* [48]. After obtaining the fundamental matrix, the essential matrix *E* can be calculated, which can be decomposed to estimate the pose of the camera [49]. The relationship between the fundamental matrix and the essential matrix can be described as follows:

(2) $$E=K^{T}FK,$$

where *K* is the intrinsic matrix of the camera of a smartphone. By utilizing singular value decomposition (SVD) [50] of *E*, the rotation matrix *R* and translation vector *T* can be calculated. The result of SVD of the essential matrix can be described as follows:

(3) $$R=\left\{\begin{array}{c} UWV^{T}\\ UW^{T}V^{T} \end{array}\right.\;T=\left\{\begin{array}{c} U{\left(0,0,1\right)}^{T}\\ -U{\left(0,0,1\right)}^{T}, \end{array}\right.$$

where *U* and *V* are the orthogonal matrices of SVD, and *W* is a constant matrix. The triangulation method [49] is used to select the correct solution from the four kinds of combinations.

According to the rotation matrix *R* of the two adjacent images, the heading angle change can be expressed by:

(4) $$R=\left[\begin{array}{ccc} cos\Delta \theta & 0 & sin\Delta \theta \\ sin\Delta \vartheta sin\Delta \theta & cos\Delta \vartheta & -sin\Delta \vartheta cos\Delta \theta \\ -cos\Delta \vartheta sin\Delta \theta & sin\Delta \vartheta & cos\Delta \vartheta cos\Delta \theta \end{array}\right],$$

where Δθ is the heading angle change of sampling point $P_{t}$ (i.e., sampled at instant *t*), and Δϑ is the pitch angle change of the sampling point. If the initial heading angle is $0^{\circ }$, the heading angle of the sampling instant can be calculated as:

(5) $$\theta_{t}=\sum_{i=1}^{t}\Delta \theta_{i},$$

where the $\theta_{t}$ is the heading angle of sampling point $P_{t}$.

### 3.3. Trajectory Recovering

The aim of trajectory recovering is to provide accurate location information for sampling points that are also candidates for Wi-Fi fingerprints. The location of a sampling point can be calculated as follows:

(6) $$\left\{\begin{array}{c} x_{t}=x_{k-1}+D\cdot sin\left(\theta_{t-1}+\Delta \theta_{t}\right),\\ y_{t}=x_{k-1}+D\cdot cos\left(\theta_{t-1}+\Delta \theta_{t}\right), \end{array}\right.$$

where ($x_{t}$, $y_{t}$) are the coordinates of sampling point $P_{t}$, $\theta_{t-1}$ is the heading angle of sampling point $P_{t-1}$, and $\Delta \theta_{t}$ is the heading angle change of $P_{t}$ that is relative to $P_{t-1}$. D is the distance between $P_{t}$ and $P_{t-1}$.

According to Equation (6), there are two types of error sources for trajectory recovery: the distance estimation error and the heading angle estimation error. In most cases, the distance estimation accuracy is not as critical as the heading angle estimation accuracy [51]. The proposed SFM-based method described in Section 3.2 provides a solution for the calculation of heading angle change (i.e., parameter Δθ in Equation (6)). However, the performance of this method is highly dependent on the results of image matching. If the matching of two adjacent sample images fails (this usually occurs if an image is of poor quality or has few distinctive features, e.g., blank walls), the estimated heading angle will be inaccurate.

To solve this problem, inertial information is employed to improve the performance of heading estimation. Similar to many PDR systems, heading angle change (Δθ) can also be calculated as the integral of the angular velocity (rad/s) with respect to time. Compared to SFM-based heading estimation, the gyroscope-based method has a higher sampling rate (more than 100 Hz), but also more drift error. Its estimation error will accumulate over time. Consequently, the gyroscope-based estimation is used as a replacement for the SFM-based estimation when the matching of adjacent images fails:

(7) $$\Delta \theta_{t}=\theta_{gyr}\;if\left(N_{t}<N_{th}\right),$$

where $\Delta \theta_{t}$ is the heading change of $P_{t}$, and $\theta_{gyr}$ is the heading change calculated from gyroscope readings. $N_{t}$ is the number of matched keypoint pairs, and $N_{th}$ is a threshold that is set to 8 in this study.

Based on the calculation of heading angle, PDR is implemented to estimate the location of each sampling point from a trajectory. A step detection method [52] is then used to estimate the distance between each pair of adjacent sampling points, based on accelerometer data. As shown in Figure 3, the timespan of each step of a walking trajectory is obtained by the use of a peak detection algorithm [53]. The length of each step can be estimated based on a frequency-based model [54]:

(8) $$step\_length_{i}=a\cdot f+b,$$

where $step\_length_{i}$ is the length of the *i*-th step of a trajectory (i.e., $step_{i}$), *f* is the step frequency, and *a* and *b* are parameters. Due to the high sampling rate, each step contains multiple sampling points. In this study, it is assumed that the sampling points within a step are equally spaced. The distance between two sampling points from a trajectory can be calculated as follows:

(9) $$distance_{j,j+1}=\frac{1}{k}step\_length_{i}\;P_{j}\;P_{j+1}\in S_{i}^{P},$$

where $P_{j}$, $P_{j+1}$ are the two adjacent sampling points that are within the *i*-th step of a trajectory, $distance_{j,j+1}$ is the distance between $P_{j}$ and $P_{j+1}$, $step\_length_{i}$ is the length of $step_{i}$, $S_{i}^{P}$ is the set of sampling points within $step_{i}$, and *k* is the number of sampling points in $S_{i}^{P}$. The coordinates of each sampling point can be calculated by using Equation (6).

### 3.4. Radio Map Construction

The attributes of the sampling points are shown in Table 1. Although these sampling points are associated with both location and RSS attributes, they cannot be directly used as Wi-Fi fingerprints. Unlike the fingerprints collected by site surveying, the sampling points from trajectories are not uniformly distributed in an indoor space. We partition the whole space into regular grids and associate each grid with both location and RSS attributes (i.e., a fingerprint). However, due to the uniform distribution and high sampling rate, it is possible that a grid contains dozens of sampling points, while another does not contain any sampling points. Moreover, the Wi-Fi scanning time of a sampling point (about 0.03 s) is much shorter than that of site surveying (usually 30–120 s), which may result in insufficient Wi-Fi scanning.

**Figure 4:**
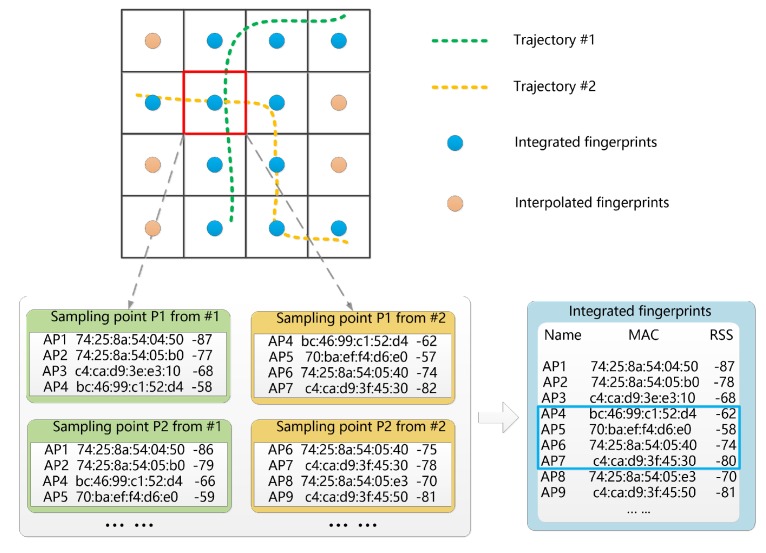
Integration of Wi-Fi APs for a fingerprint.

To solve these problems, the fingerprints in this study are generated based on integrating the received signal strength (RSS) of the sampling points. Similar to many fingerprinting approaches, an indoor space is partitioned into regular grids. Each grid is treated as a fingerprint that is located at its center. As shown in Figure 4, the RSS of a fingerprint is calculated by combining the RSS of sampling points (from one or more trajectories) within its spatial extent:

(10) $$FAP^{i}=\cup_{j\in G_{i}}AP^{j},$$

where $FAP^{i}$ is the set of access points (APs) for fingerprint *i*, $AP^{j}$ is the set of APs for sampling point *j*, $G_{i}$ is the set of sampling points for fingerprint *i* (i.e., within the spatial extent of grid *i*). The RSS of fingerprint *i* can be calculated as follows:

(11) $$RSS_{j}\left(i\right)=\frac{1}{n}\sum_{k\in G_{i}}RSS_{j}^{k},$$

where $RSS_{j}\left(i\right)$ is the RSS of AP *j* in $FAP^{i}$, $G_{i}$ is the set of sampling points for fingerprint *i*, $RSS_{j}^{k}$ is the $RSS_{j}$ (i.e., the RSS of the *j*-th AP) of the *k*-th sampling point for fingerprint *i*, and *n* is the number of sampling points for fingerprint *i*. Note that $RSS_{j}^{k}$ equals 0 if AP *j* is not an AP member of sampling point *k*. If a fingerprint does not have any sampling points, the Wi-Fi APs, as well as the RSS value, can be calculated by an interpolation method [55]. The first step for calculating the interpolated fingerprint is to construct the set of APs, according to its nearest fingerprints. We select the intersection of APs within its 4-neighborhood as the interpolated Wi-Fi APs:

(12) $$IFAP^{i}=\cap_{i\in N_{i}}FAP^{i},$$

where $IFAP^{i}$ is the set of APs for interpolated fingerprint *i*, and $N_{i}$ is the set of neighborhood fingerprints used for interpolation. The RSS of interpolated fingerprint *i* can be calculated using the inverse distance weight function, which can be described as:

(13) $$w\left(x\right)=e^{-ax},$$

where the constant *a* is a positive value. The interpolation function can be expressed as follows:

(14) $$RSS\left(i\right)=\frac{\sum_{j}w\left(d_{j}\right)\cdot RSS\left(N_{j}\right)}{\sum_{j}w\left(d_{j}\right)},$$

where RSS(i) is the RSS of the interpolated fingerprint *i*, $d_{j}$ is the distance between fingerprint *j* and fingerprint *i*. $RSS(N_{j})$ is the RSS of the set of neighbored fingerprints.

The integration of sampling points can enrich the RSS information of a fingerprint, which alleviates the problem of the short Wi-Fi scanning time of sampling points. To further improve the quality of the generated fingerprints for indoor localization, the outliers should be removed from the RSS of the fingerprints. Here, an outlier is defined as the RSS of an AP, which is not accurate for the corresponding fingerprint. Outliers may be caused by either the location estimation error of sampling points or the fluctuation of Wi-Fi signals. Based on the standard deviation of the RSS, the threshold for outlier determination can be calculated as follows:

(15) $$Thr_{j}^{i}=m\cdot \frac{1}{n}\sum_{k=0}^{n}\left|RSS_{j}^{k}-RSS_{j}\left(i\right)\right|,$$

where $Thr_{j}^{i}$ is the RSS threshold of AP *j* for fingerprint *i*, $RSS_{j}^{k}$ is the $RSS_{j}$ of the *k*-th sampling point for fingerprint *i*, $RSS_{j}\left(i\right)$ is the RSS of AP *j* in $AP^{i}$, *n* is the number of sampling points for fingerprint *i*, and *m* is a parameter that is set to 2.5 in this study. If the RSS of AP *j* is outside the range of $Thr_{j}^{i}$ from $RSS_{j}\left(i\right)$, it is treated as an outlier for fingerprint *i*. The RSS of the fingerprints is recalculated after removing all the outliers. The generated fingerprints constitute the radio map for indoor localization. The constructed radio map can be updated constantly with the increase of trajectory data.

## 4. Evaluations

### 4.1. Experiment Setup

**Figure 5:**
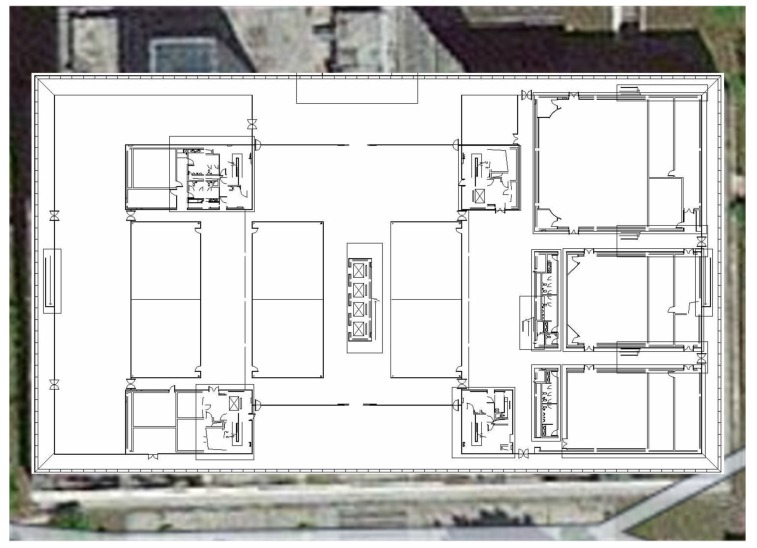
Layout of the study area.

In this section, we conducted three experiments on the ground floor of the Science and Technology Building, Shenzhen University, Shenzen, China. As depicted in Figure 5, this area spans an area of 106 × 61 m and contains both wide areas and narrow corridor areas. An Android version 4.3 Galaxy Note 3 smartphone (SAMSUNG, Korea,2013) was used to collect experiment data, including Wi-Fi RSS, inertial data and video frames. During data collection, the sampling frequency of corresponding sensors were about 250 HZ, 100 HZ and 30 fps.

The first experiment aimed to evaluate the performance of the heading angle estimation method. During this experiment, a smartphone was vertically fixed on an Edmund Optics (East Gloucester Pike, Barrington) rotary stage and was rotated around the *z*-axis of the rotary stage at different angle changes. The smartphone collected both video frames and gyroscope data during the process, which were used to estimate the heading angles using the proposed method. In addition, the collected gyroscope data was used alone to estimate the same heading angles for comparison: the angles were calculated as the integral of the angular velocity (rad/s) with respect to time. The second experiment evaluated the performance of the trajectory recovering method. During the experiment, participants held a smartphone in front of them (keeping the camera forward facing and maintaining the posture) and walked at a normal pace in the public space of the study area. It is assumed that the walking mode of the participant will not change from walking to running (or jogging). The built-in sensors of the smartphone (Galaxy Note 3) collected the experiment data including video frames, inertial sensor data and Wi-Fi signals for recovering the trajectories of participants. We define the difference between the viewing direction of the camera and the walking direction of the participant as the heading offset. If the heading offset is large, the area of overlap between a pair of adjacent frames may be small, which may lead to the failure of image matching. In this study, the heading offset of less than $10^{\circ }$ can be tolerated without difficulty to image matching and SFM. There is no constraint for turning activities of the participants. Similar to the first experiment, the collected inertial data, including acceleration and gyroscope readings, was used alone to recover the same trajectories for comparison. The heading angles were calculated using the gyroscope data (the integral of the angular velocity with respect to time) and the travelled distances were estimated using the PDR method described in Section 3.3. To verify the performance of the visual-based approach, the third experiment was implemented to test the quality of the constructed radio map for indoor localization.

### 4.2. Performance of Heading Angle Estimation

The estimation of heading angle change (i.e., turning angle) is a core question for trajectory recovery. We tested the proposed heading angle estimation method with the experience that the angle between adjacent frames is no more than $20^{\circ }$. During the experiment, a smartphone was vertically fixed on an Edmund Optics rotary stage and was rotated around the *z*-axis of the rotary stage at three different angles ($5^{\circ }$, $10^{\circ }$, $15^{\circ }$ and $20^{\circ }$). The rotation angle could be obtained directly from the dials of the rotary stage. For each rotation angle ($5^{\circ }$, $10^{\circ }$, $15^{\circ }$ and $20^{\circ }$), the rotation of the smartphone was repeated 20 times. Consequently, 80 videos were collected by the smartphone camera. The turning angles of these rotations were estimated by two different methods: (1) the gyroscope-based method; (2) the visual/inertial integrated method. The estimation errors of the heading angle change were evaluated as follows:

(16) $$A_{err}=\frac{1}{n}\sum_{i}^{n}\left|A_{j}^{E}-A_{i}^{G}\right|,$$

where $A_{err}$ is the mean error of the estimations for a type of rotation, $A_{i}^{E}$ is the estimated heading angle change of the *i*-th rotation, $A_{i}^{G}$ is the actual heading angle change of the *i*-th ground-truth point, and *n* is the number of rotations.

**Figure 6:**
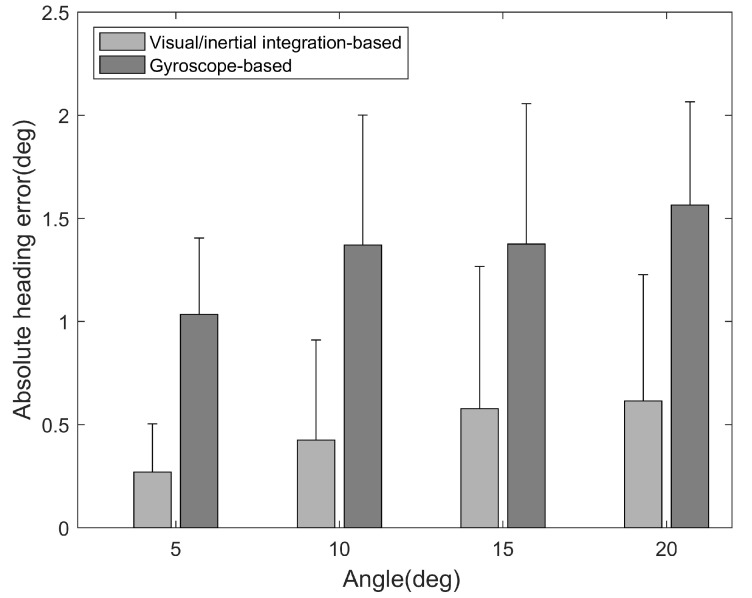
The errors of two heading angle estimation methods.

Figure 6 showed the heading estimation error of the two methods at four angular intervals. The $A_{err}$ of the gyroscope-based method ($1.03^{\circ }$ for $5^{\circ }$; $1.37^{\circ }$ for $10^{\circ }$; $1.38^{\circ }$ for $15^{\circ }$; $1.56^{\circ }$ for $20^{\circ }$) is obviously higher than that of the visual/inertial integration-based method ($0.27^{\circ }$ for $5^{\circ }$; $0.42^{\circ }$ for $10^{\circ }$; $0.57^{\circ }$ for $15^{\circ }$; $0.61^{\circ }$ for $20^{\circ }$). The maximum error of heading estimation is lower than $2.5^{\circ }$, the mean error is lower than $0.7^{\circ }$, and 80 percent error of the heading angle is below $0.5^{\circ }$. It indicates that this method performs well under different rotation angle conditions and can be used to estimate the azimuth of walking trajectory.

### 4.3. Performance of Trajectory Restoring

**Figure 7:**
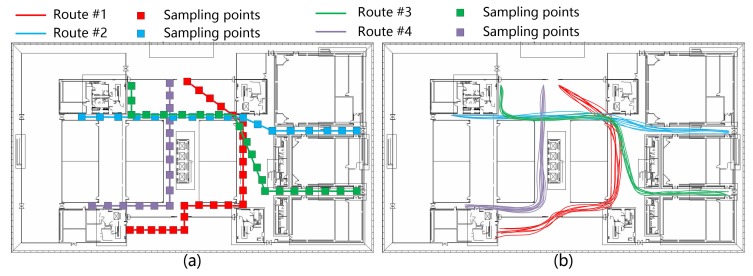
Four represented routes to verify the proposed trajectory restoring method. (**a**) is the ground truth data; (**b**) is the restored trajectories using the proposed method.

In order to verify the accuracy of this trajectory recovering method, two participants (one male and one female) were asked to walk along four routes with known initial locations, as shown in Figure 7a. Each route was repeated 10 times by the participants. Before the experiment, all the trajectories were uniformly sampled to obtain a sequence of ground-truth points. During the experiment, the smartphones were held by the participants and kept forward facing at a fixed posture to collect the inertial and video data continuously. A student recorded the times when participants walked past each marker. Images of the sampling points were extracted from the video frames. The heading angle of each sampling point was calculated by the visual/inertial integration-based method. The distance between adjacent sampling points was estimated based on the step detection method. The reconstruction results of the trajectories are shown in Figure 7b.The overall error of all the trajectories is 0.53 m (SD = 0.4 m), which represents the average distance between each pair of estimated sampling point and its corresponding ground-truth point.

**Figure 8:**
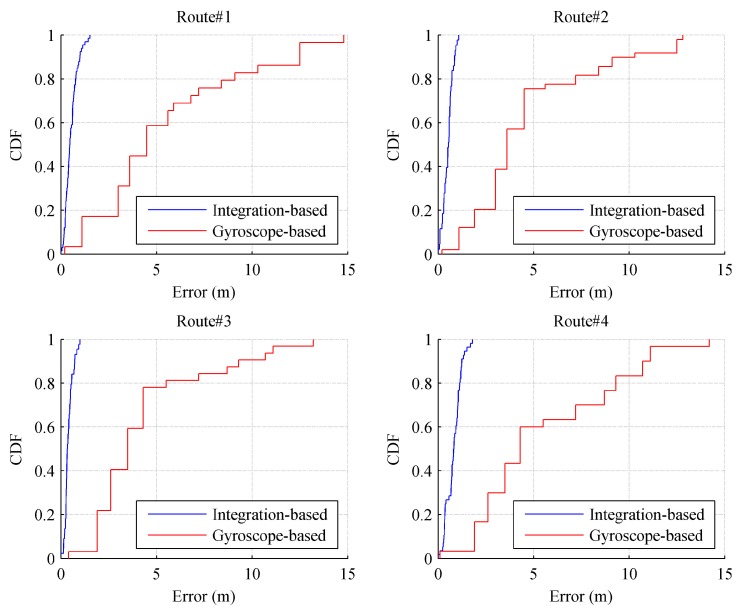
The quantitative results of annotation errors.

The shape discrepancy metric (SDM) was used as a metric to quantify the difference between the shapes of the recovered trajectories and the real ones. In [56], the SDM is defined as the Euclidean distance between a sampling point and its corresponding ground-truth point. Figure 8 shows the cumulative distribution function (CDF) of the SDM for 40 trajectories using a visual/inertial integration-based method and the gyroscope-based method. Clearly, the SDM error of the gyroscope-based method is much higher than that of the integration-based methods. For the integration-based method, the maximum SDM error is about 1.5 m; the 80-percentile SDM error is around 1 m; and the mean SDM error is about 0.53 m. This result indicates that visual information can help to improve the location accuracy of the trajectory recovery. It also demonstrates that the integration of both visual and inertial information helps to overcome the drawbacks of single-source based methods, e.g., drift error from the gyroscope or matching failure of the SFM. Furthermore, the experimental trajectories covered wide spaces in the study area. This approach performs well in wide indoor space, which increases the potential for applying it to large indoor environments (e.g., shopping malls).

### 4.4. Performance of Indoor Localization

**Figure 9:**
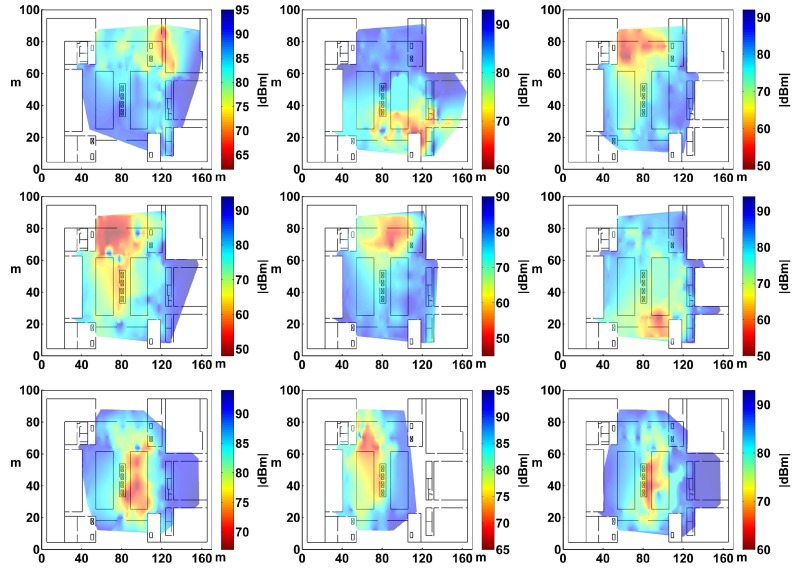
The visual results of radio maps.

To construct a radio map, another 100 trajectories were collected and recovered that covers most of the public area in the study area. There were mainly three steps. First, the study area was partitioned into a 2.4 m × 2.4 m mesh grid. Then, the collected trajectories (that generally covered the public space of the study area) were used to generate fingerprints and construct radio maps by using the proposed method (described in Section 3.4). The generated fingerprints located at the center of the corresponding grids. Figure 9 shows the visual results of different APs from the constructed radio map. Finally, the quality of the constructed radio map was compared with another radio map constructed by site surveying, which was conducted at the center of the same grids. An online localization experiment was conducted based on the weighted k-nearest neighbor method using the two radio maps, respectively. In the experiment, the online RSS measurements were collected at the center of 60 grids (the same spots as the reference points in the radio map). The localization error was calculated as follows:

(17) $$Err_{i}=\sqrt{{\left(x_{i}^{r}-x_{i}^{e}\right)}^{2}+{\left(y_{i}^{r}-y_{i}^{e}\right)}^{2}},$$

where $Err_{i}$ is the localization error of point *i*, ($x_{i}^{r}$, $y_{i}^{r}$) is the actual physical location of point *i*, and ($x_{i}^{e}$, $y_{i}^{e}$) is the estimated physical location of point *i*.

**Figure 10:**
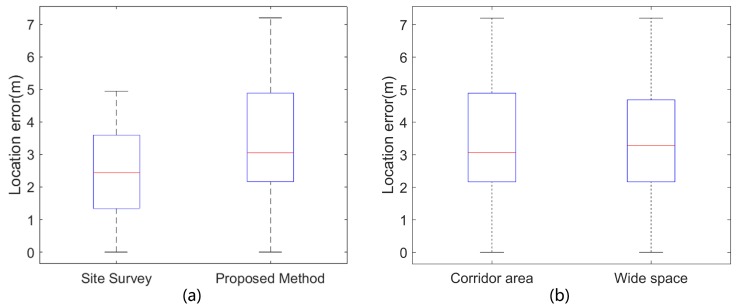
Localization performance of the proposed method. (**a**) The localization error of two methods; (**b**) The localization error of the proposed method in two difference indoor spaces.

The localization results of two methods are shown in Figure 10a. The site survey method achieved a relatively higher accuracy. The average localization error of the site survey method is slightly smaller than that of the proposed method (3.2 m). It indicates that the quality of the constructed radio map is at the same level as the site survey-based radio map. Figure 10b shows that the proposed method achieves similar results (average location error) in two different types of environments: corridors (about 3.2 m) and wide spaces (about 3.4 m). It demonstrates that this method can be applied to both corridor-like spaces and wide spaces. The freedom of walking direction is quite high in wide spaces, which limits the application of map matching based localization methods. By integrating both visual and inertial information, this method can significantly improve the performance of trajectory recovery and provide accurate location labels for WiFi fingerprints, which are important to the generation of high-quality radio maps.

In summary, the visual-based approach can provide indoor radio maps of similar quality with that collected by site surveys. However, this can greatly reduce the human labor needed for fingerprints collection. Moreover, it performs well in wide indoor spaces, which increases the potential for applying this approach to large indoor environments such as shopping malls, underground parking garages, or supermarkets.

## 5. Conclusions

In this study, a visual-based approach was proposed for the automatic construction of indoor radio maps. It could accurately restore indoor walking trajectories and calibrate Wi-Fi fingerprints by using the built-in sensors of smartphones. A visual/inertial integration-based method was developed for the estimation of heading angle. A multi-constrained image matching method was also proposed to reduce the mismatching of the SFM method and improve the accuracy of heading angle estimation. The Wi-Fi fingerprints could be extracted from the recovered trajectories for the generation of radio maps. The experiment results demonstrated that the visual-based trajectory restoring method was able to provide accurate location labels for WiFi fingerprints. The quality of constructed radio map is at the same level as the site survey based radio map. This approach has the potential to be applied to large indoor environments for effective collection of radio maps. In future work, we will improve the localization algorithm used in this approach and apply it to various indoor environments.

References

## Figures and Tables

**Table 1:** The attributes of the sampling points.

Sampling Point ID	Time	Trajectory ID	AP	Coordinates	RSS
p1	t1	Tr_1	{$ap_{1}$, $ap_{2}$...}	($X_{1}$, $Y_{1}$)	{$rss_{1}$, $rss_{2}$...}
p2	t2	Tr_2	{$ap_{1}$, $ap_{2}$...}	($X_{2}$, $Y_{2}$)	{$rss_{1}$, $rss_{2}$...}
p3	t3	Tr_3	{$ap_{1}$, $ap_{2}$...}	($X_{3}$, $Y_{3}$)	{$rss_{1}$, $rss_{2}$...}

## References

[1] Bahl P., Padmanabhan V.N. RADAR: An in-building RF-based user location and tracking system. Proceedings of the Nineteenth Annual Joint Conference of the IEEE Computer and Communications Societies (INFOCOM).

[2] Bargh M.S., Groote R.D. Indoor localization based on response rate of bluetooth inquiries. Proceedings of the ACM International Workshop on Mobile Entity Localization and Tracking in Gps-Less Environments.

[3] Subbu K.P., Gozick B., Dantu R. (2013). LocateMe: Magnetic-fields-based indoor localization using smartphones. ACM Trans. Intell. Syst. Technol..

[4] Hazas M., Hopper A. (2006). Broadband ultrasonic location systems for improved indoor positioning. IEEE Trans. Mob. Comput..

[5] Ni L.M., Liu Y., Lau Y.C., Patil A.P. (2004). LANDMARC: Indoor location sensing using active RFID. Wirel. Netw..

[6] Fontana R.J., Gunderson S.J. Ultra-wideband precision asset location system. Proceedings of the 2002 IEEE Conference on Ultra Wideband Systems and Technologies.

[7] Zhou J., Chu M.K., Ng K.Y. Providing location services within a radio cellular network using ellipse propagation model. Proceedings of the International Conference on Advanced Information Networking and Applications.

[8] Raspopoulos M., Laoudias C., Kanaris L., Kokkinis A. Cross device fingerprint-based positioning using 3D Ray Tracing. Proceedings of the 2012 8th International Wireless Communications and Mobile Computing Conference (IWCMC).

[9] Sorour S., Lostanlen Y., Valaee S., Majeed K. (2013). Joint indoor localization and radio map construction with limited deployment load. IEEE Trans. Mob. Comput..

[10] Bolliger P. Redpin—Adaptive, zero-configuration indoor localization through user collaboration. Proceedings of the ACM International Workshop on Mobile Entity Localization and Tracking in Gps-Less Environments.

[11] Yang S., Dessai P., Verma M., Gerla M. FreeLoc: Calibration-free crowdsourced indoor localization. Proceedings of the 2013 Proceedings IEEE INFOCOM.

[12] Wu C., Yang Z., Liu Y., Xi W. (2013). WILL: Wireless indoor localization without site survey. IEEE Trans. Parallel Distrib. Syst..

[13] Wu C., Yang Z., Liu Y. (2014). Smartphones based crowdsourcing for indoor localization. IEEE Trans. Mob. Comput..

[14] Yu N., Xiao C., Wu Y., Feng R. (2016). A radio-map automatic construction algorithm based on crowdsourcing. Sensors.

[15] Youssef M., Agrawala A. The Horus WLAN location determination system. Proceedings of the International Conference on Mobile Systems, Applications, and Services.

[16] Castro P., Chiu P., Kremenek T., Muntz R. A probabilistic room location service for wireless networked environments. Proceedings of the 3rd International Conference on Ubiquitous Computing.

[17] Roos T., Myllymäki P., Tirri H., Misikangas P., Sievänen J. (2002). A probabilistic approach to WLAN user location estimation. Int. J. Wirel. Inf. Netw..

[18] Park J.G., Charrow B., Curtis D., Battat J., Minkov E., Hicks J., Teller S., Ledlie J. Growing an organic indoor location system. Proceedings of the International Conference on Mobile Systems, Applications, and Services.

[19] Au A.W.S., Feng C., Valaee S., Reyes S., Sorour S., Markowitz S.N., Gold D., Gordon K., Eizenman M. (2013). Indoor tracking and navigation using received signal strength and compressive sensing on a mobile device. IEEE Trans. Mob. Comput..

[20] Pratama A.R., Hidayat R. Smartphone-based Pedestrian Dead Reckoning as an indoor positioning system. Proceedings of the International Conference on System Engineering and Technology.

[21] Gusenbauer D., Isert C., Krösche J. Self-contained indoor positioning on off-the-shelf mobile devices. Proceedings of the International Conference on Indoor Positioning and Indoor Navigation.

[22] Link J.A.B., Smith P., Viol N., Wehrle K. FootPath: Accurate map-based indoor navigation using smartphones. Proceedings of the International Conference on Indoor Positioning and Indoor Navigation.

[23] Wang H., Sen S., Elgohary A., Farid M., Youssef M., Choudhury R.R. No need to war-drive: Unsupervised indoor localization. Proceedings of the International Conference on Mobile Systems, Applications, and Services.

[24] House S., Connell S., Milligan I., Austin D., Hayes T.L., Chiang P. Indoor localization using pedestrian dead reckoning updated with RFID-based fiducials. Proceedings of the 2011 Annual International Conference of the IEEE Engineering in Medicine and Biology Society.

[25] Chen Z., Zou H., Jiang H., Zhu Q., Soh Y.C., Xie L. (2015). Fusion of WiFi, smartphone sensors and landmarks using the Kalman filter for indoor localization. Sensors.

[26] Zhou B., Li Q., Mao Q., Tu W., Zhang X. (2015). Activity sequence-based indoor pedestrian localization using smartphones. IEEE Trans. Hum.-Mach. Syst..

[27] Gluckman J., Nayar S.K. Ego-motion and omnidirectional cameras. Proceedings of the International Conference on Computer Vision.

[28] Irani M., Rousso B., Peleg S. Recovery of ego-motion using image stabilization. Proceedings of the 1994 IEEE Conference on Computer Vision and Pattern Recognition.

[29] Olson C.F., Matthies L.H., Schoppers M., Maimone M.W. (2003). Rover Navigation using Stereo Ego-motion. Robot. Auton. Syst..

[30] Milella A., Siegwart R. Stereo-based ego-motion estimation using pixel tracking and iterative closest point. Proceedings of the IEEE International Conference on Computer Vision Systems.

[31] Porzi L., Ricci E., Ciarfuglia T.A., Zanin M. Visual-inertial tracking on Android for Augmented Reality applications. Proceedings of the IEEE Workshop on Environmental Energy and Structural Monitoring Systems (EESMS).

[32] Klein G., Murray D. Parallel tracking and mapping for small AR workspaces. Proceedings of the IEEE and ACM International Symposium on Mixed and Augmented Reality.

[33] Mohatta S., Perla R., Gupta G., Hassan E., Hebbalaguppe R. Robust hand gestural interaction for smartphone based AR/VR applications. Proceedings of the IEEE Winter Conference in Applications of Computer Vision.

[34] He H., Li Y., Guan Y., Tan J. (2015). Wearable ego-motion tracking for blind navigation in indoor environments. IEEE Trans. Autom. Sci. Eng..

[35] Fang W., Zheng L., Deng H. A motion tracking method by combining the IMU and camera in mobile devices. Proceedings of the International Conference on Sensing Technology.

[36] Li Y., Wang S., Yang D., Sun D. (2016). A novel metric online monocular SLAM approach for indoor applications. Sci. Progr..

[37] Ruotsalainen L., Kuusniemi H., Chen R. Heading change detection for indoor navigation with a Smartphone camera. Proceedings of the International Conference on Indoor Positioning and Indoor Navigation.

[38] Ruotsalainen L., Kuusniemi H., Bhuiyan M.Z., Chen L., Chen R. (2013). A two-dimensional pedestrian navigation solution aided with a visual gyroscope and a visual odometer. GPS Solut..

[39] Lacroix S., Mallet A., Chatila R., Gallo L. (1999). Rover self localization in planetary-like environments. Artif. Intell..

[40] Dong J., Xiao Y., Noreikis M., Ou Z., Jaaski A.Y. iMoon: Using smartphones for image-based indoor navigation. Proceedings of the ACM Conference on Embedded Networked Sensor Systems.

[41] Kim H., Lee D., Oh T., Choi H.T., Myung H. (2015). A probabilistic feature map-based localization system using a monocular camera. Sensors.

[42] Jung S.H., Taylor C.J. Camera trajectory estimation using inertial sensor measurements and structure from motion results. Proceedings of the 2001 IEEE Computer Society Conference on Computer Vision and Pattern Recognition.

[43] Hakeem A., Vezzani R., Shah M., Cucchiara R. Estimating geospatial trajectory of a moving camera. Proceedings of the 18th International Conference on Pattern Recognition.

[44] Lowe D.G. (2004). Distinctive image features from scale-invariant keypoints. Int. J. Comput. Vis..

[45] Ledwich L., Williams S. Reduced SIFT features for image retrieval and indoor localisation. Proceedings of the Australian Conference on Robotics and Automation.

[46] Fischler M.A., Bolles R.C. (1981). Random sample consensus: A paradigm for model fitting with applications to image analysis and automated cartography. Commun. ACM.

[47] Bouguet J.Y. Camera Calibration Toolbox for Matlab. http://www.vision.caltech.edu/bouguetj/calib_doc/.

[48] Luong Q.T., Faugeras O.D. (1996). The fundamental matrix: Theory, algorithms, and stability analysis. Int. J. Comput. Vis..

[49] Hartley R. (2003). Multiple View Geometry in Computer Vision.

[50] Golub G.H., Reinsch C. (1970). Singular value decomposition and least squares solutions. Numer. Math..

[51] Chen W., Chen R., Chen Y., Kuusniemi H., Wang J. An effective Pedestrian Dead Reckoning algorithm using a unified heading error model. Proceedings of the IEEE/ION Position, Location and Navigation Symposium.

[52] Alzantot M., Youssef M. UPTIME: Ubiquitous pedestrian tracking using mobile phones. Proceedings of the Wireless Communications and Networking Conference.

[53] Mladenov M., Mock M. A step counter service for Java-enabled devices using a built-in accelerometer. Proceedings of the 1st International Workshop on Context-Aware Middleware and Services: Affiliated with the 4th International Conference on Communication System Software and Middleware.

[54] Cho D.K., Min M., Lee U., Kaiser W.J. AutoGait: A mobile platform that accurately estimates the distance walked. Proceedings of the Eigth IEEE International Conference on Pervasive Computing and Communications.

[55] Ezpeleta S., Claver J.M., Pérezsolano J.J., Martí J.V. (2015). RF-Based Location Using Interpolation Functions to Reduce Fingerprint Mapping. Sensors.

[56] Shen G., Chen Z., Zhang P., Moscibroda T., Zhang Y. Walkie-Markie: Indoor pathway mapping made easy. Proceedings of the Usenix Conference on Networked Systems Design and Implementation.

